# Supplementary material for: nMOWChIP-seq: low-input genome-wide mapping of non-histone targets
Source: NAR Genom Bioinform. 2022 Apr 7;4(2):lqac030. doi: 10.1093/nargab/lqac030 (PMC8988714; doi:10.1093/nargab/lqac030)
Supplement: lqac030_Supplemental_Files [file lqac030_supplemental_files.zip › Supplementary figures and tables_2-21-2022_final.pdf]

Supplementary figures and tables

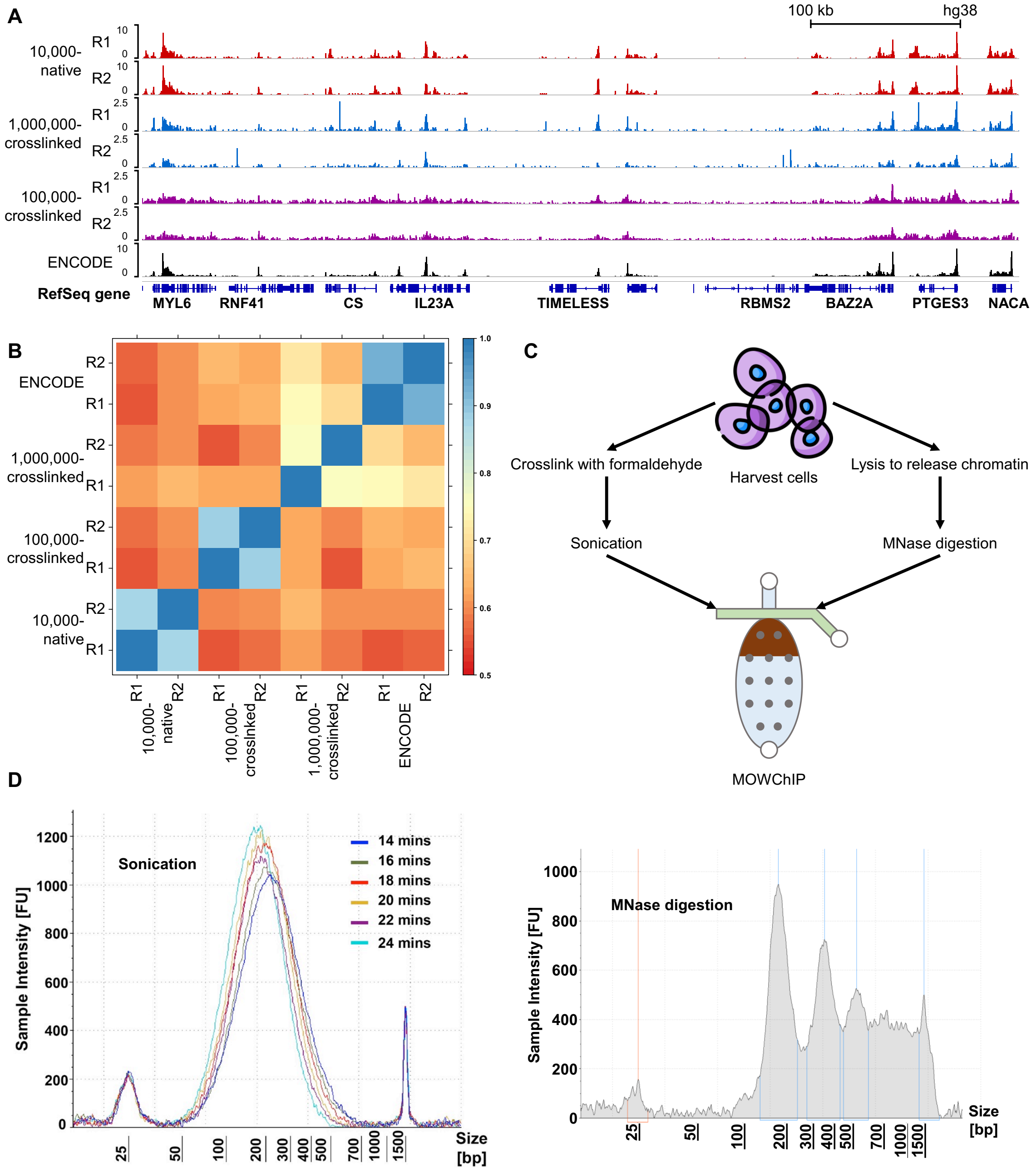

Supplementary Figure S1. Comparison of MNase-based native MOWChIP-seq with crosslinked MOWChIP-seq. (A) Normalized Pol II (S5) signal of GM12878 using MOWChIP-seq, with either native (10,000 cells per assay) or crosslinked (1 million and 100,000 cells per assay) chromatin. (B) Pearson's correlation matrix of native and crosslinked MOWChIP-seq data and ENCODE data (SRX100530), computed using DiffBind affinity score method. (C) Illustration showing the procedural difference between crosslinked and native MOWChIP. (D) Size distribution of chromatin after various durations of sonication. 16 min was selected as the optimal duration. Size distribution of MNase-digested chromatin is shown as comparison on the right.

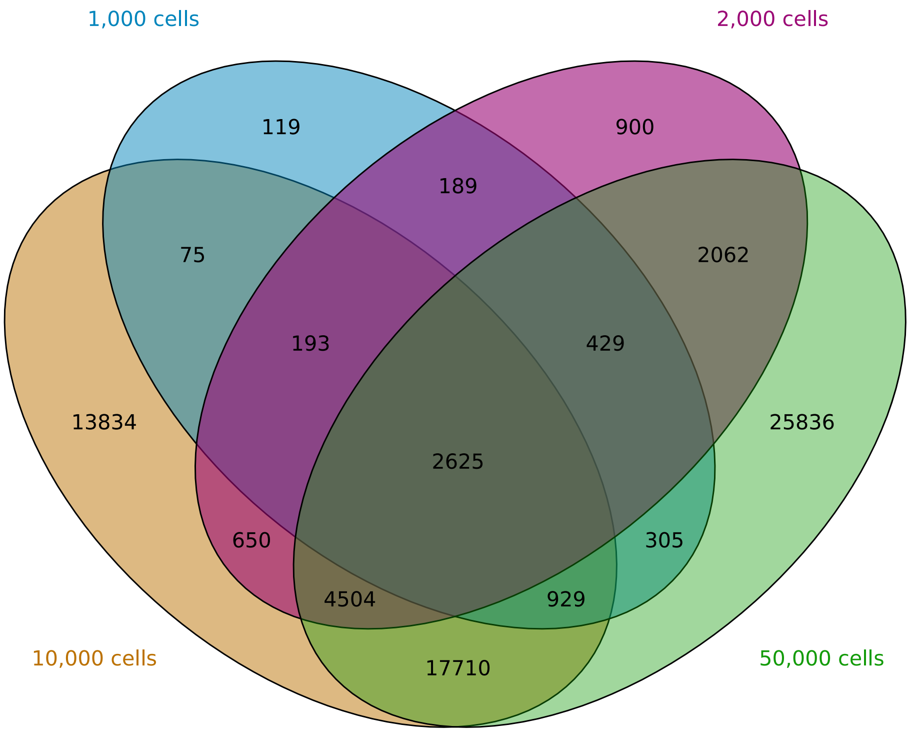

Supplementary Figure S2. Overlap of Pol II-S5 peaks among assays using 50,000, 10,000, 2,000 and 1,000 cells per assay. Peaks from technical replicates are checked with bedtools and those overlap more than 50% are merged to generate a peak set for the condition. Overlap among the 4 peak sets are plotted using Intervene.

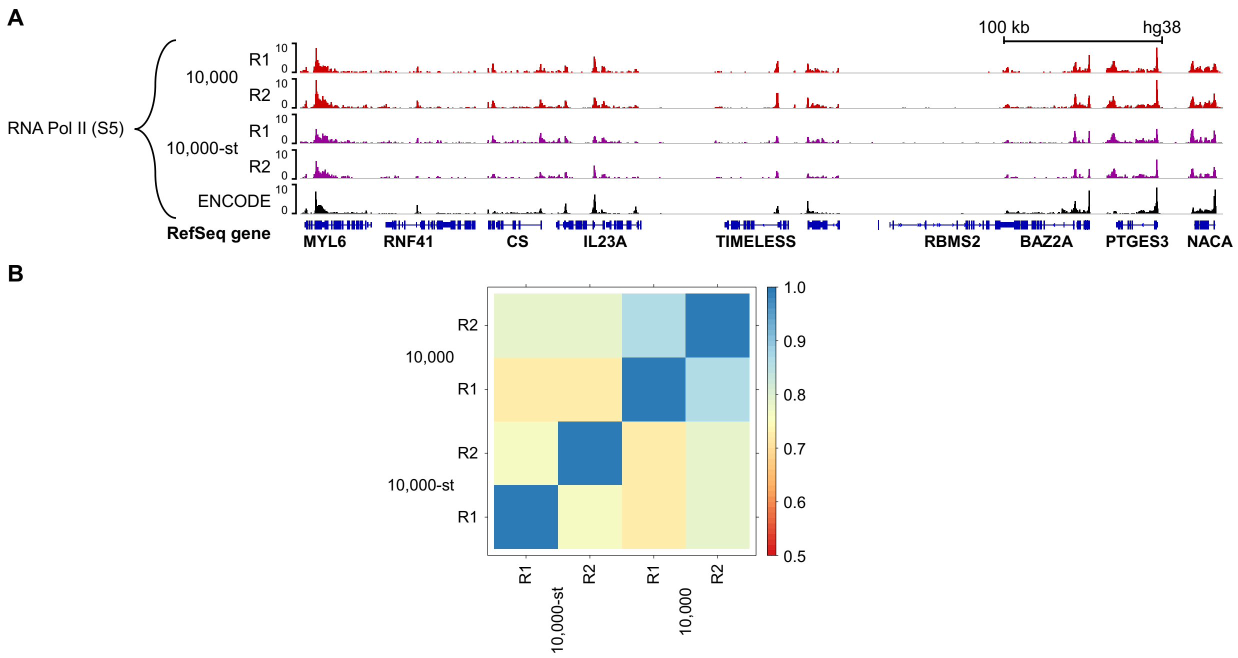

Supplementary Figure S3. Storage at -80 °C does not change RNA pol II binding profile. (A) Normalized Pol II-S5 signal of GM12878 cells. "10,000-st" denotes chromatin samples (each containing chromatin fragments from 10,000 cells) that were stored at -80 °C for 2 d and then used for nMOWChIP-seq. "10,000" refers to samples processed without such storage. In contrast, we found that freezing of chromatin samples at -80 °C substantially varies TF binding profiles. (B) Pearson's correlation matrix of Pol II-S5 nMOWChIP-seq data of fresh and -80 °C stored GM12878 chromatin, computed using DiffBind affinity score method.

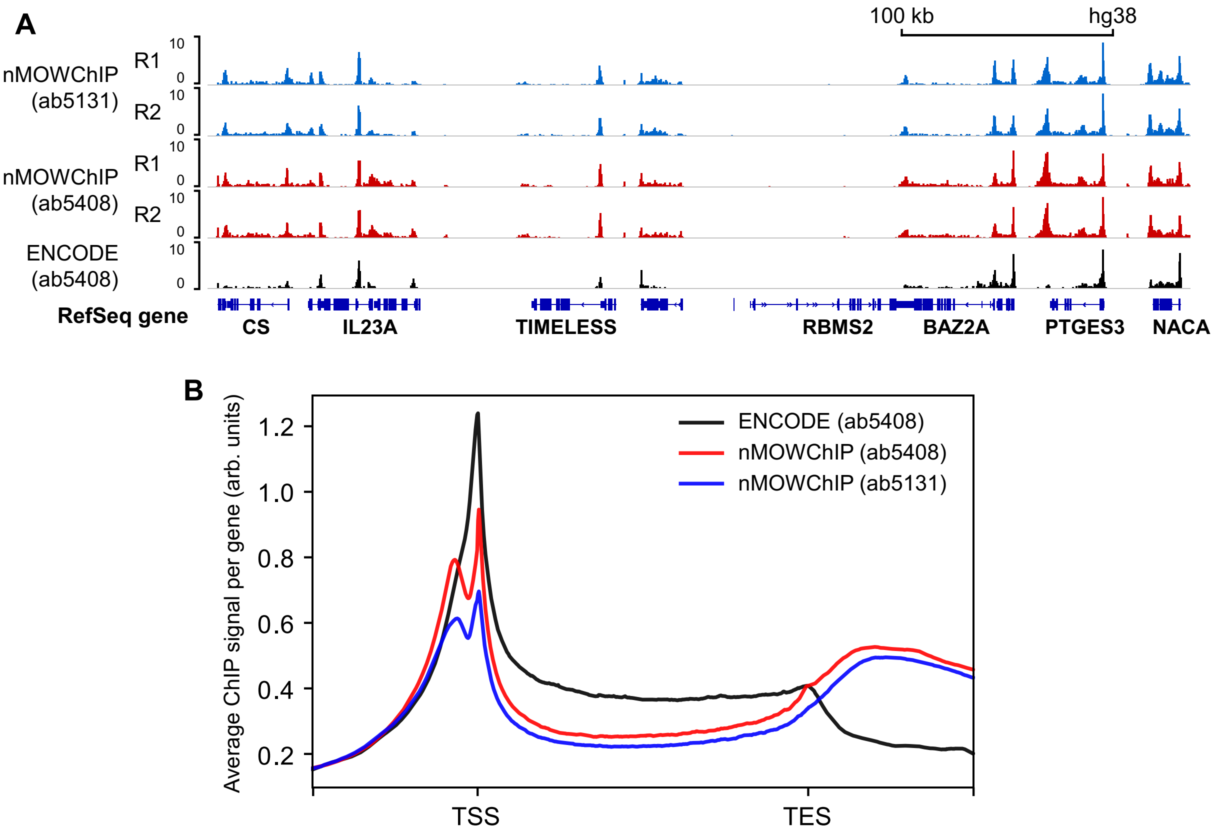

Supplementary Figure S4. Comparison of nMOWChIP-seq data and ENCODE data produced using conventional ChIP-seq technology. GM12878 cells were used in all experiments. (A) Normalized Pol II-S5 signals generated with Pol II-S5 antibody ab5131 (Abcam, rabbit polyclonal) and ab5408 (Abcam, mouse monoclonal 4H8), using nMOWChIP (50,000 cells per assay). ENCODE data was generated with antibody ab5408 (crosslinked, 10 million cells per assay). (B) Average ChIP-seq signal per gene over promoter and gene body for nMOWChIP-seq and ENCODE data. Averagely higher signal near TES regions could be observed in nMOWChIP-seq data with both antibodies.

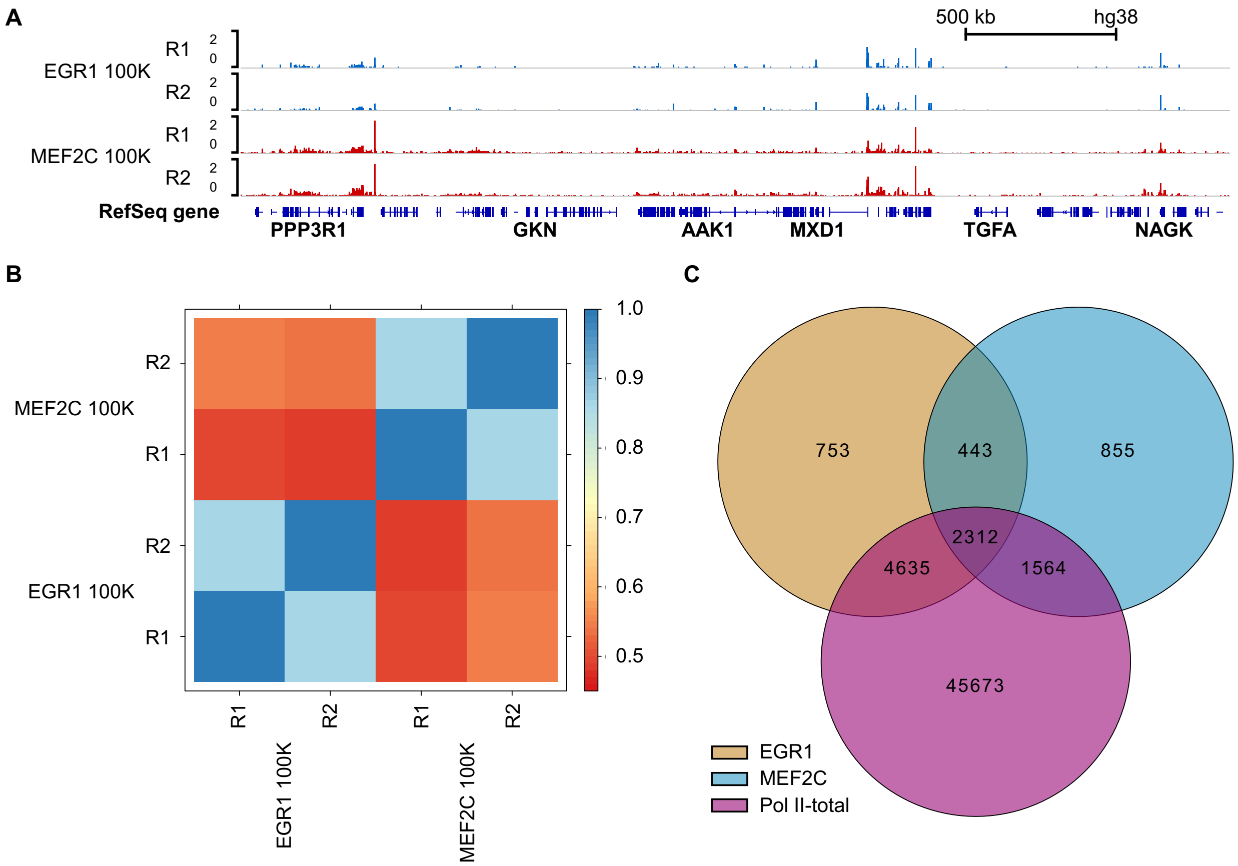

Supplementary Figure S5. MEF2C and EGR1 data on GM12878 using 100,000 cells per assay. (A) Normalized signal of EGR1 and MEF2C generated using 100,000 GM12878 cells. (B) Correlation matrix computed using DiffBind, showing relatively low correlation between the two transcription factors in GM12878 (~0.52). (C) Overlap of peak sets among EGR1, MEF2C and Pol II-total in GM12878 cells. Peaks were identified by MACS2 using default settings. Peaks from both replicates that overlap more than 50% of their length are merged to generate a peak set using bedtools. The overlap among the peak sets was plotted using Intervene.

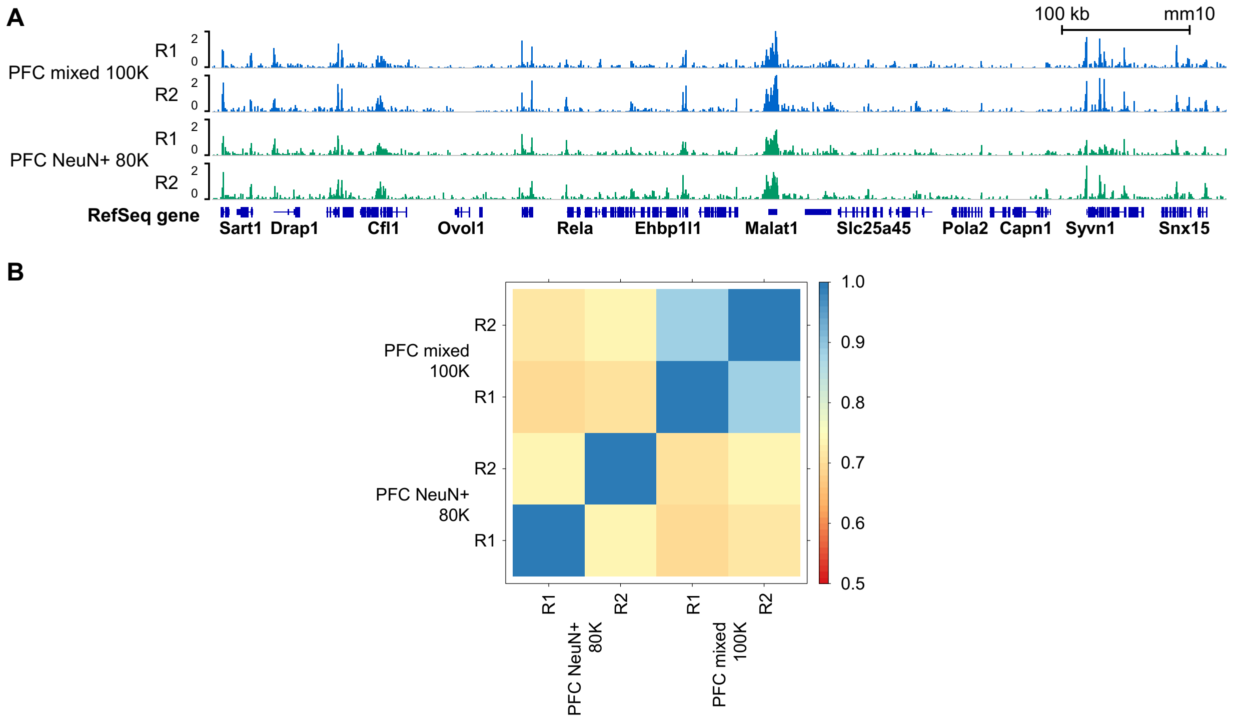

Supplementary Figure S6. HDAC2 binding in mouse PFC samples profiled by nMOWChIP-seq. (A) Normalized HDAC2 signals generated using nuclei from mouse PFC. 100,000 unsorted nuclei from PFC, and 80,000 of FACS-sorted NeuN+ (neuronal) nuclei were used per assay in these tests. (B) Pearson's correlation matrix on HDAC2 mouse PFC data, computed using DiffBind affinity score method.

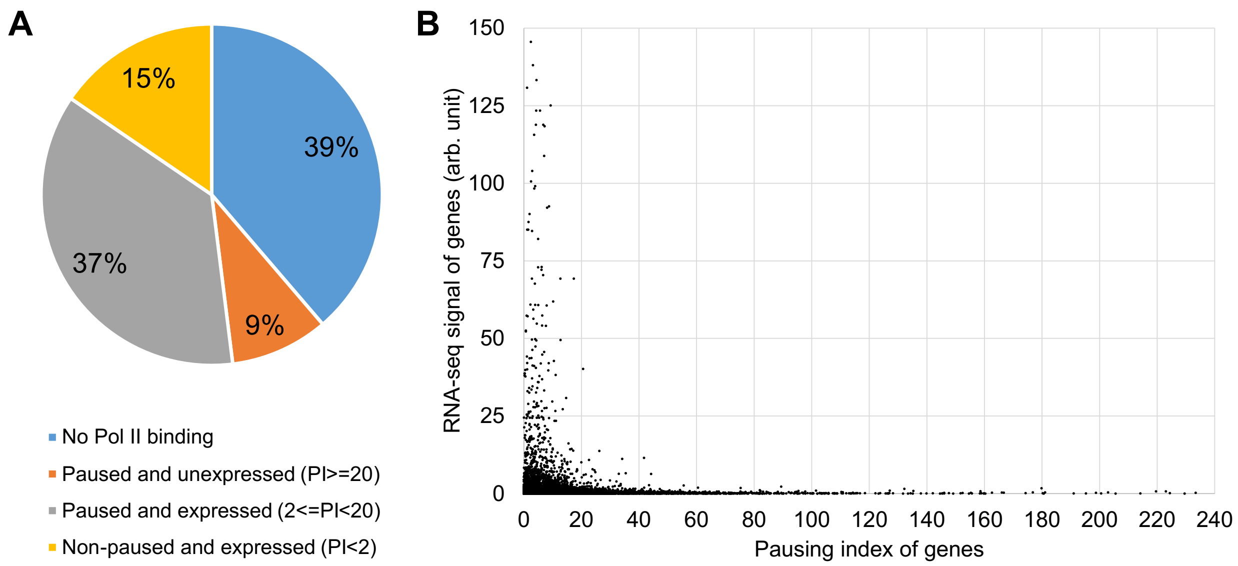

Supplementary Figure S7. Distribution of pausing index (PI) for genes (A) in GM12878 cells and the relationship between PI and RNA-seq signal of the gene (B) based on Pol II-total nMOWChIP-seq data. RNA-seq data were obtained from ENCODE (ENCSR000AEH).

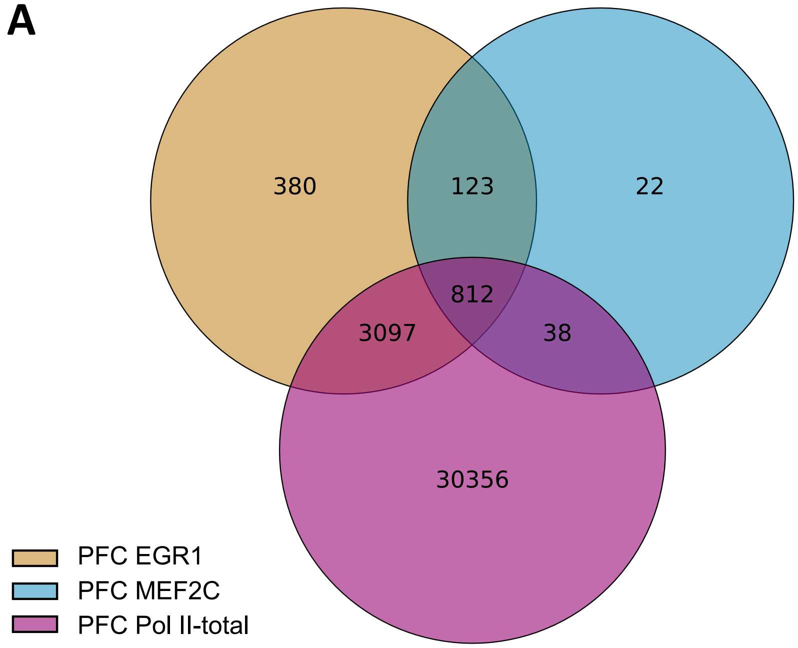

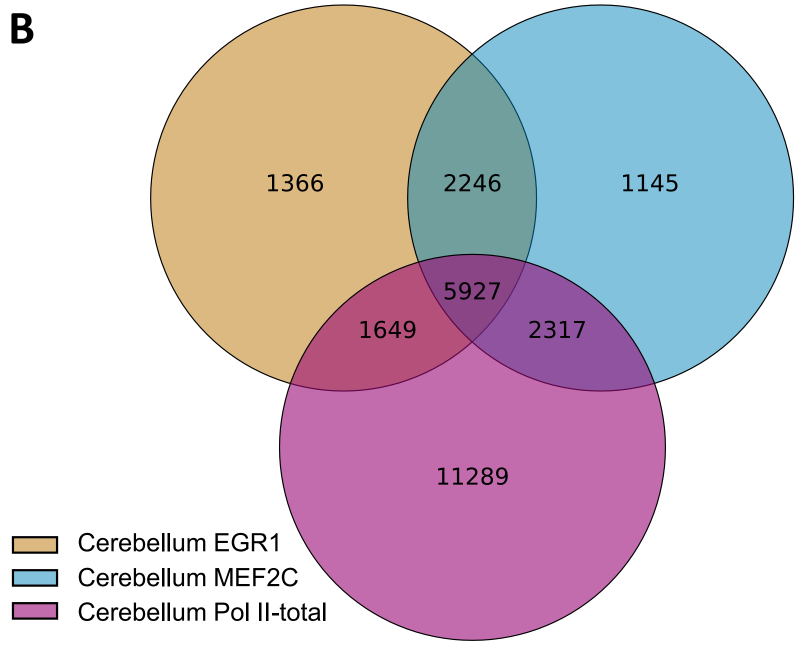

Supplementary Figure S8. Overlap among Pol II, EGR1, and MEF2C peaks in mouse PFC (A) and cerebellum (B) data. Peaks were identified by MACS2 using default settings. Peaks from both replicates that overlap more than 50% of their length are merged to generate a peak set using bedtools. The overlap of the peak sets was plotted using Intervene.

Supplementary Table S1. Summary of nMOWChIP-seq data and ENCODE data for comparison, including number and rate of reads mapped, number and rate of reads that mapped to one unique location on the genome, number of peaks called by MACS2, and fraction of reads that falls within the range of called peaks (FRiP). FRiP values are calculated after subsampling all the datasets to 9 million raw reads.

File	Mapped reads	Mapping Rate	Unique reads	Unique rate	Number of peaks	FRiP
GM_Pol2-total_50K_1	16432424	98%	16204517	99%	133015	49.6%
GM_Pol2-total_50K_2	20219467	98%	19820603	98%	129219	47.6%
GM_Pol2-S5-4H8_50K_1	12458138	94%	12275183	99%	144609	52.4%
GM_Pol2-S5-4H8_50K_2	13400605	94%	13231849	99%	152047	52.3%
GM_Pol2-S5_50K_1	16006273	98%	15570715	97%	142339	44.0%
GM_Pol2-S5_50K_2	15203135	98%	14377380	95%	130669	42.5%
GM_Pol2-S5_10K_1	12271356	96%	12061753	98%	114811	37.3%
GM_Pol2-S5_10K_2	21780356	96%	21550914	99%	140695	40.7%
GM_Pol2-S5_10K_st_1	9646207	95%	9354458	97%	82320	26.6%
GM_Pol2-S5_10K_st_2	13775137	96%	13338787	97%	79800	22.5%
GM_Pol2-S5_2K_1	14243331	90%	13432201	94%	59713	13.4%
GM_Pol2-S5_2K_2	15242929	87%	14400694	94%	70439	19.0%
GM_Pol2-S5_1K_1	15572762	87%	14923018	96%	33627	9.8%
GM_Pol2-S5_1K_2	9194036	86%	8777057	95%	19820	7.5%
GM_Pol2-S5_100K_cl_1	31745569	98%	31406005	99%	53817	3.5%
GM_Pol2-S5_100K_cl_2	31111394	98%	30545134	98%	17561	0.6%
GM_EGR1_100K_1	14323523	96%	12199195	85%	16614	7.2%
GM_EGR1_100K_2	17458334	96%	14961426	86%	15584	6.8%
GM_EGR1_50K_1	14910367	95%	12800282	86%	16465	7.7%
GM_EGR1_50K_2	16669698	95%	14480504	87%	15159	7.4%
GM_EGR1_10K_1	9072849	84%	7725182	85%	2626	3.6%
GM_EGR1_10K_2	12461029	94%	10785293	87%	2781	2.3%
GM_EGR1_5K_1	10330104	92%	8383719	81%	8245	4.4%
GM_EGR1_5K_2	12660454	93%	11175451	88%	1423	1.6%
GM_MEF2C_100K_1	24369737	76%	21133894	87%	14716	3.1%
GM_MEF2C_100K_2	24642860	85%	20705206	84%	19884	5.1%
GM_HDAC2_100K_1	21068312	84%	18801502	89%	11221	2.7%
GM_HDAC2_100K_2	19862691	85%	18127467	91%	5832	1.7%
GM_HDAC2_50K_1	8767036	93%	8093468	92%	1204	1.0%
GM_HDAC2_50K_2	12215492	93%	11358639	93%	1584	0.9%
PFC_mix_Pol2-total_50K_1	13064208	97%	12803650	98%	66123	45.6%
PFC_mix_Pol2-total_50K_2	16395371	99%	16251965	99%	80138	50.4%
PFC_mix_Pol2-total_10K_1	8574476	95%	7748982	90%	24423	24.6%
PFC_mix_Pol2-total_10K_2	13007858	93%	12338679	95%	53291	41.7%

Cerebellum_mix_Pol2-total_50K_1	10617272	99%	10444570	98%	43387	50.2%
Cerebellum_mix_Pol2-total_50K_2	9805738	98%	9553592	97%	36762	46.2%
Cerebellum_mix_Pol2-total_10K_1	15483210	97%	15200342	98%	54534	63.9%
Cerebellum_mix_Pol2-total_10K_2	14308273	98%	14139656	99%	54278	67.8%
PFC_EGR1_100K_1	9285737	91%	8104052	87%	6212	9.5%
PFC_EGR1_100K_2	8557087	51%	7510446	88%	6921	10.1%
Cerebellum_EGR1_100K_1	9882524	95%	8739064	88%	17795	14.7%
Cerebellum_EGR1_100K_2	13141514	95%	11799729	90%	22866	12.4%
PFC_MEF2C_100K_1	8478934	82%	7497951	88%	1354	7.1%
PFC_MEF2C_100K_2	11407867	91%	10012928	88%	5362	8.0%
Cerebellum_MEF2C_100K_1	11268711	95%	10016208	89%	24063	16.5%
Cerebellum_MEF2C_100K_2	11481247	95%	10025093	87%	17393	14.1%
PFC_HDAC2_100K_1	14011639	85%	11164030	80%	2929	13.1%
PFC_HDAC2_100K_2	15354891	85%	12429080	81%	3465	12.2%
PFC_Neuron_HDAC2_80K_1	12258720	80%	10188120	83%	1382	10.7%
PFC_Neuron_HDAC2_80K_2	12448796	80%	10639071	85%	2842	8.8%
ENCODE_GM_Pol2-4H8_1 (SRX100530)	33028003	92%	32625068	99%	104405	30.9%
ENCODE_GM_Pol2-4H8_2 (SRX100530)	28400615	95%	28109992	99%	118295	37.6%
ENCODE_GM_Pol2-8WG16_1 (SRX100400)	23018947	72%	22857329	99%	116251	46.2%
ENCODE_GM_Pol2-8WG16_2 (SRX100400)	14324178	65%	14223930	99%	136649	44.5%
ENCODE_GM_EGR1_1 (SRX100479)	21822180	94%	21424699	98%	60826	6.1%
ENCODE_GM_EGR1_2 (SRX100479)	27455047	96%	26888014	98%	29439	7.2%
ENCODE_GM_HDAC2_1 (SRX3322296)	24504181	94%	23632278	96%	3540	0.2%
ENCODE_GM_HDAC2_2 (SRX3322297)	34555254	94%	33249270	96%	100	0.1%

Supplementary Table S2. List of genes with significant difference in pausing index between PFC and cerebellum based on mouse brain Pol II-total data (fold change > 3, minimal read density > 0.02, p-value < 0.05, FDR < 0.05)

Cerebellum > PFC				
Gene	Fold change	Minimum read density	p-value	FDR
Plk2	10.2	0.187	7.94E-04	1.89E-03
Npas4	10.0	0.151	2.36E-03	4.95E-03
Rec8	7.8	0.043	2.18E-02	2.12E-02
Snapc2	7.7	0.108	1.90E-03	4.02E-03
Nsg1	7.2	0.044	4.60E-06	4.40E-05
Slc1a2	7.1	0.021	1.10E-02	1.48E-02
Grik5	6.8	0.021	4.65E-02	2.83E-02
Camk2n1	6.7	0.119	2.57E-04	7.70E-04
Sox12	6.7	0.099	4.44E-05	1.65E-04
Dos	6.5	0.129	2.21E-02	2.13E-02
Chtf18	6.4	0.050	2.05E-03	4.35E-03
Sept5	6.2	0.104	1.62E-02	1.85E-02
Tro	6.0	0.030	1.82E-03	3.91E-03
Barhl2	6.0	0.031	2.46E-02	2.25E-02
Gm2506	6.0	0.023	7.13E-04	1.72E-03
Syt7	5.7	0.025	2.76E-02	2.35E-02
Zfp296	5.7	0.061	1.42E-02	1.72E-02
Gpr123	5.6	0.021	1.03E-03	2.39E-03
Arpp19	5.4	0.057	2.66E-03	5.43E-03
Nfyb	5.4	0.028	6.58E-04	1.50E-03
Rapgefl1	5.3	0.059	1.42E-04	4.73E-04
Lmo4	5.1	0.057	7.41E-03	1.13E-02
Arc	5.1	0.200	1.08E-02	1.45E-02
Celf5	5.0	0.045	2.17E-03	4.54E-03
Tubb2a	5.0	0.116	1.35E-04	4.40E-04
Ephx4	4.9	0.025	6.43E-03	1.01E-02
Tmem151a	4.9	0.073	7.91E-05	2.64E-04
Nap1l1	4.9	0.029	2.27E-02	2.16E-02
Gpc1	4.8	0.029	4.24E-02	2.73E-02
Ino80e	4.8	0.052	1.93E-02	2.01E-02
Gnmt	4.7	0.021	4.80E-03	8.39E-03
Kif17	4.7	0.030	2.22E-05	9.89E-05
Emilin3	4.6	0.031	4.36E-02	2.76E-02
Mtch1	4.6	0.046	9.31E-03	1.32E-02
Arpp21	4.6	0.041	3.68E-04	9.34E-04
Ptprn	4.5	0.075	6.06E-03	9.83E-03
Rgs2	4.5	0.180	9.53E-05	3.52E-04
Kcnt1	4.5	0.024	1.72E-02	1.89E-02

Stk19	4.5	0.038	1.97E-03	4.17E-03
BC018242	4.3	0.069	8.81E-03	1.27E-02
Klhdc8a	4.3	0.028	1.80E-04	5.83E-04
A930018M24Rik	4.2	0.112	9.98E-03	1.39E-02
Sun2	4.1	0.028	7.04E-03	1.08E-02
Prkag1	4.0	0.027	1.07E-03	2.46E-03
Pigz	3.9	0.051	1.56E-03	3.41E-03
Zdhhc8	3.9	0.032	8.07E-04	1.92E-03
Man2a2	3.9	0.027	6.80E-03	1.05E-02
Rgs4	3.9	0.113	4.07E-03	7.39E-03
Ppp1r1a	3.9	0.098	1.69E-03	3.68E-03
Micall1	3.9	0.034	5.68E-03	9.34E-03
Rasd1	3.8	0.057	2.72E-03	5.56E-03
Tacc1	3.8	0.023	1.64E-05	6.60E-05
Rtn3	3.8	0.024	2.64E-02	2.30E-02
Ncan	3.8	0.057	1.99E-02	2.03E-02
Gm996	3.7	0.031	4.81E-02	2.86E-02
Gria2	3.7	0.025	2.48E-02	2.25E-02
Trim9	3.7	0.025	3.28E-05	1.32E-04
Arhgdia	3.7	0.059	2.07E-03	4.39E-03
Nrn1	3.7	0.061	5.56E-03	9.26E-03
Srm	3.7	0.041	3.05E-03	6.09E-03
Vipr1	3.6	0.021	7.37E-04	1.78E-03
Gm10116	3.6	0.032	9.18E-04	2.10E-03
Fam49a	3.6	0.020	7.64E-04	1.86E-03
Gm4535	3.6	0.054	2.74E-02	2.33E-02
Ttyh3	3.5	0.028	1.33E-04	4.18E-04
BC025920	3.5	0.026	3.67E-03	6.96E-03
Gnao1	3.5	0.020	2.16E-02	2.11E-02
Ltk	3.5	0.024	3.02E-02	2.43E-02
Ywhaz	3.5	0.078	2.10E-02	2.09E-02
Xrcc3	3.5	0.024	3.44E-02	2.54E-02
Tceb2	3.5	0.070	1.68E-02	1.88E-02
Sh3glb2	3.5	0.039	1.19E-03	2.73E-03
1700088E04Rik	3.4	0.091	6.70E-03	1.04E-02
Camk2n2	3.4	0.030	4.27E-02	2.73E-02
Arpc2	3.4	0.020	2.75E-03	5.60E-03
C030046I01Rik	3.4	0.062	4.95E-03	8.62E-03
Cacnb1	3.4	0.047	1.53E-02	1.79E-02
1110008P14Rik	3.4	0.106	5.51E-04	1.29E-03
Gal3st3	3.3	0.026	1.71E-02	1.89E-02
Usp30	3.3	0.025	2.12E-02	2.09E-02
Ccdc157	3.3	0.021	4.28E-04	1.07E-03

Dpysl2	3.3	0.028	9.39E-05	3.30E-04
Trim46	3.2	0.063	2.74E-02	2.34E-02
Atp1a3	3.2	0.092	7.12E-03	1.09E-02
R3hdm1	3.2	0.021	7.65E-04	1.87E-03
Eme1	3.2	0.029	9.86E-03	1.37E-02
Aifm3	3.2	0.030	7.95E-03	1.19E-02
Asphd1	3.2	0.076	5.71E-03	9.39E-03
Mcl1	3.2	0.089	3.00E-03	6.04E-03
Ppil2	3.2	0.033	1.09E-02	1.47E-02
Josd1	3.1	0.044	3.93E-04	9.89E-04
Cdc42ep3	3.1	0.027	4.04E-02	2.68E-02
Mcrs1	3.1	0.051	4.45E-04	1.10E-03
Bloc1s2	3.1	0.035	8.16E-03	1.22E-02
Rhog	3.1	0.029	3.99E-02	2.67E-02
Klhdc8b	3.1	0.029	1.28E-02	1.62E-02
BC017643	3.1	0.076	9.99E-03	1.39E-02
Rnf139	3.1	0.026	5.84E-03	9.61E-03
Jdp2	3.1	0.020	1.10E-03	2.54E-03
4931428F04Rik	3.1	0.057	2.28E-03	4.75E-03
Slc4a3	3.1	0.079	1.23E-03	2.83E-03
Efhd2	3.1	0.051	5.42E-03	9.12E-03
Pcgf2	3.1	0.028	5.64E-04	1.30E-03
Rab35	3.1	0.022	2.76E-02	2.35E-02
1700019L03Rik	3.1	0.030	1.12E-02	1.49E-02
Hexdc	3.1	0.024	2.50E-03	5.18E-03
Tcea2	3.0	0.060	4.29E-05	1.54E-04
Dusp7	3.0	0.027	2.60E-02	2.29E-02
Pdlim7	3.0	0.041	7.78E-03	1.18E-02
Pacsin3	3.0	0.034	1.29E-02	1.62E-02
Epn3	3.0	0.032	1.01E-02	1.40E-02
Sepw1	3.0	0.093	9.61E-04	2.21E-03
PFC > Cerebellum				
Gene	Fold change	Minimum read density	p-value	FDR
Dlk2	3.0	0.056	4.52E-03	7.96E-03
Rps6ka1	3.0	0.021	2.98E-03	5.96E-03
Mycl1	3.1	0.024	1.14E-02	1.50E-02
Carhsp1	3.2	0.042	1.99E-02	2.03E-02
Shank3	3.2	0.022	4.03E-02	2.68E-02
Sparcl1	3.2	0.025	4.65E-05	1.87E-04
Fgfbp3	3.2	0.024	2.41E-02	2.23E-02
Slc16a8	3.2	0.030	5.33E-03	9.04E-03
Gpsm3	3.3	0.029	6.04E-03	9.80E-03
Smtn	3.3	0.033	2.10E-02	2.09E-02

Fosb	3.5	0.070	1.02E-02	1.40E-02
Trip6	3.5	0.024	3.54E-03	6.75E-03
Flrt2	3.6	0.025	7.57E-03	1.16E-02
Cul5	3.6	0.022	1.33E-02	1.65E-02
Gm11696	3.7	0.030	1.78E-02	1.93E-02
Cbln1	3.7	0.108	2.09E-03	4.40E-03
E130303B06Rik	3.7	0.044	1.11E-02	1.48E-02
Proca1	4.1	0.035	3.17E-02	2.47E-02
Pak6	4.1	0.024	2.98E-02	2.41E-02
Gpt	4.3	0.047	5.80E-03	9.53E-03
Lrg1	4.3	0.030	3.26E-02	2.50E-02
Btbd17	4.5	0.037	3.78E-02	2.62E-02
Pnrc1	4.8	0.024	2.39E-02	2.22E-02
Ascl1	5.1	0.043	2.25E-02	2.15E-02
Kank2	5.3	0.020	2.92E-03	5.82E-03
Tcte1	5.3	0.024	2.53E-02	2.27E-02
Eif4e	5.3	0.024	1.84E-02	1.96E-02
2610203C20Rik	5.3	0.039	1.80E-02	1.94E-02
Chrm4	5.5	0.051	1.23E-02	1.57E-02
Ramp2	5.6	0.034	7.13E-03	1.09E-02
A230052G05Rik	5.7	0.024	1.52E-02	1.78E-02
0610007L01Rik	5.7	0.036	4.37E-02	2.76E-02
Hrc	5.7	0.067	1.40E-02	1.71E-02
Doc2a	6.0	0.073	2.35E-02	2.20E-02
Rsph9	6.0	0.024	1.81E-02	1.95E-02
Npy1r	6.2	0.025	1.43E-02	1.72E-02
Eno3	6.2	0.038	1.45E-02	1.74E-02
Scn3b	6.4	0.031	2.51E-03	5.19E-03
Ntn3	6.4	0.058	3.20E-02	2.48E-02
Ndrg4	6.4	0.085	4.81E-03	8.40E-03
Shroom1	6.6	0.028	4.54E-02	2.80E-02
Dusp18	6.6	0.033	1.76E-02	1.92E-02
Loxl3	6.6	0.028	3.26E-02	2.50E-02
Ttc19	6.9	0.028	1.56E-02	1.81E-02
Dnaja3	7.1	0.023	4.79E-03	8.38E-03
Def6	7.1	0.020	2.47E-03	5.13E-03
Plch2	7.2	0.035	2.94E-03	5.86E-03
Bdh1	7.3	0.021	2.42E-02	2.23E-02
Nol3	8.1	0.088	5.23E-03	8.93E-03
Coch	8.4	0.024	7.53E-03	1.15E-02
Gm20537	8.7	0.021	4.83E-02	2.86E-02
Acta1	8.8	0.073	1.27E-03	2.86E-03
Tmem88b	9.1	0.089	1.34E-02	1.67E-02

Cox6b2	9.5	0.027	1.71E-02	1.89E-02
Fam164c	9.8	0.026	2.25E-02	2.15E-02
BC106179	10.2	0.027	8.09E-03	1.21E-02
S1pr5	11.2	0.031	1.72E-02	1.89E-02
Ntpcr	12.3	0.024	1.31E-02	1.64E-02
Msl3l2	12.8	0.045	4.33E-02	2.74E-02
Arpc1b	13.2	0.025	3.72E-04	9.56E-04
Slc16a3	13.6	0.023	5.00E-02	2.90E-02
Pqlc3	14.1	0.025	8.24E-04	1.97E-03
1700019N12Rik	17.0	0.024	4.68E-03	8.20E-03
Set	21.3	0.021	1.23E-02	1.56E-02
Kank3	35.0	0.099	9.79E-03	1.37E-02
Mad2l2	42.0	0.049	9.94E-03	1.38E-02
Acyp1	77.4	0.030	1.66E-02	1.87E-02

Supplementary Table S3. Primers used in qPCR enrichment tests of nMOWChIP-seq libraries.

Species	Locus name	Primer
Human (GM12878 cells)	*ACTG1* (F)	CGG AAA GAT CGC CAT ATA TGG AC
	ACTG1(R)	ACC GGC AGA GAA ACG CGA
	POLR2A (F)	GAG AGA CAA ACT GCC GTA ACC
	POLR2A (R)	GGG AAA TAA GGA GCG AAA GGA G
	AFM (F)	GCA GAA CCT AGT TCC TCC TTC AAC
	AFM (R)	AGT CAT CCC TTC CTA CAG ACT GAG A
	KCTD16 (F)	GCC ATA AAG AAA CAC ACA TGG AAA C
	KCTD16 (R)	GCA AAG CAG GAA CCC TCA AA
Mouse brain (PFC and cerebellum)	*Arc* (F)	CAG CAT AAA TAG CCG CTG GT
	Arc (R)	GTC GCC GCT GAA GCT AGA
	Polr2a (F)	AAA GAA GGG AGG AGA GGA GGA
	Polr2a (R)	GGG AGA GAC AAA CTG CCG TAA
	Afm (F)	CAT TTG ACC CAA ACT GCT AAG T
	Afm (R)	GTA TGT GAA GTG TCA GCA ATG G
	Gcg (F)	TCA ACC CAA AGT CCC TGA AG
	Gcg (R)	CCA AGA GGT TGC ATT GGA AG
